# Sacituzumab govitecan plus platinum-based chemotherapy mediates significant antitumor effects in triple-negative breast, urinary bladder, and small-cell lung carcinomas

**DOI:** 10.18632/oncotarget.28559

**Published:** 2024-02-22

**Authors:** Thomas M. Cardillo, Maria B. Zalath, Roberto Arrojo, Robert M. Sharkey, Serengulam V. Govindan, Chien-Hsing Chang, David M. Goldenberg

**Affiliations:** ^1^Immunomedics, Inc., Morris Plains, NJ 07950 now acquired by Gilead Sciences, Inc., Foster City, CA 94404, USA; ^2^Gilead Sciences, Inc., Foster City, CA 94404, USA; ^3^Current address: Center for Molecular Medicine and Immunology, Mendham, NJ 07945, USA; E-mail, dmg.gscancer@att.net.; ^*^At the time the work was conducted, all the authors were employees of Immunomedics, Inc.; ^#^At the time the work was conducted, this author was Chairman and Chief Scientific Officer of Immunomedics, Inc., which he founded in 1982

**Keywords:** sacituzumab govitecan, Trop-2, SN-38, carboplatin, cisplatin

## Abstract

Sacituzumab govitecan (SG) is an antibody-drug conjugate composed of an anti-Trop-2-directed antibody conjugated with the topoisomerase I inhibitory drug, SN-38, via a proprietary hydrolysable linker. SG has received United States Food and Drug Administration (FDA) approval to treat metastatic triple-negative breast cancer (TNBC), unresectable locally advanced or metastatic hormone receptor (HR)-positive, human epidermal growth factor receptor 2 (HER2)-negative breast cancer, and accelerated approval for metastatic urothelial cancer. We investigated the utility of combining SG with platinum-based chemotherapeutics in TNBC, urinary bladder carcinoma (UBC), and small-cell lung carcinoma (SCLC). SG plus carboplatin or cisplatin produced additive growth-inhibitory effects *in vitro* that trended towards synergy. Immunoblot analysis of cell lysates suggests perturbation of the cell-cycle and a shift towards pro-apoptotic signaling evidenced by an increased Bax to Bcl-2 ratio and down-regulation of two anti-apoptotic proteins, Mcl-1 and survivin. Significant antitumor effects were observed with SG plus carboplatin in mice bearing TNBC or SCLC tumors compared to all controls (*P* < 0.0062 and *P* < 0.0017, respectively) and with SG plus cisplatin in UBC and SCLC tumor-bearing animals (*P* < 0.0362 and *P* < 0.0001, respectively). These combinations were well tolerated by the animals. Combining SG with platinum-based chemotherapeutics demonstrates the benefit in these indications and warrants further clinical investigation.

## INTRODUCTION

Sacituzumab govitecan (SG) is an anti-human trophoblast cell-surface antigen-2 (Trop-2)-directed antibody-drug conjugate (ADC) comprised of a humanized IgG1-κ monoclonal antibody, hRS7, with nM affinity for human Trop-2, conjugated with an SN-38 drug-payload [1–3]. Trop-2 is a 46 KDa transmembrane glycoprotein highly expressed on many different solid tumors, including triple-negative breast cancer (TNBC), urothelial cancer (UC), and small-cell lung cancer (SCLC) [2, 4]. SN-38, a topoisomerase I (TOP1)-inhibitor and active metabolite of irinotecan, is conjugated to hRS7 via the proprietary CL2A hydrolysable linker with a drug to antibody ratio (DAR) of 7.6 [1, 3]. SG affects cells through delivery of its SN-38 payload to Trop-2-expressing tumors followed by rapid release of SN-38 inside the cell allowing for killing via double-stranded DNA breaks (dsDNA) of both targeted cells and bystander tumor cells [5, 6].

An early SG clinical trial (NCT01631552) demonstrated efficacy and safety in several different solid tumor disease indications including metastatic TNBC (mTNBC), metastatic UC (mUC), SCLC, metastatic non-small-cell lung carcinoma (mNSCLC), and endometrial cancer [7–13]. In the pivotal SG phase III ASCENT confirmatory trial (NCT02574455), mTNBC patients that received at least one prior therapy for metastatic disease, responded to SG with significant improvement in progression-free survival (PFS) and overall survival (OS) compared to single-agent chemotherapy of physician’s choice [14]. Likewise, in the currently active TROPHY-U-01 phase II, multicohort, open label, registration trial, patients with mUC previously treated with platinum-based and/or checkpoint inhibitors received SG therapy (NCT03547973). Initial results from the first cohort of 113 patients with a median follow-up of 9.1 months, show an ORR of 27% with 77% of the patients demonstrating a decrease in measurable disease [15]. Based on these clinical data, SG recently gained regular approval by the United States Food and Drug Administration (FDA) in mTNBC patients who received two or more prior systemic therapies, at least one of them for metastatic disease. Most recently, based on the results of TROPICS-02 clinical study (NCT03901339), FDA approved SG for unresectable locally advanced or metastatic hormone receptor (HR)-positive, human epidermal growth factor receptor 2 (HER2)-negative breast cancer (mHR^+^/HER2^-^ BC) in patients that have progressed after endocrine-based therapy and at least two additional systemic therapies. Further, SG gained accelerated approval in mUC for patients with locally advanced or metastatic disease, who previously received a platinum-containing chemotherapy and either a programmed death receptor-1 (PD-1) or a programmed death-ligand 1 (PD-L1) inhibitor.

Using multiple drugs to treat cancer may allow for direct activity against multiple targets simultaneously or may indirectly affect the same target through different mechanisms of action [16]. Our first efforts into the potential advantage of combining SG with other drugs centered on poly(ADP-ribose) polymerase inhibitors (PARPi’s) [17]. We demonstrated that SG, when combined with PARPi’s, mediated greater DNA damage to TNBC tumor cells producing synergistic growth-inhibition and significantly greater antitumor effects in tumor-bearing mice with no appreciable toxicity to the animals [17]. Results of these pre-clinical studies led to clinical testing of SG combined with the PARPi rucaparib [18] (NCT03992131) and in the current clinical trial with talazoparib, in patients with mTNBC (NCT04039230), with successful completion of the dose-escalation phase 1b portion of the trial [19]. Others also demonstrated that combining TOP1 inhibitors (e.g., irinotecan, SN-38, or topotecan) with platinum-based chemotherapeutics produced synergistic growth-inhibitory effects *in vitro* in a variety of human tumor lines, including hematopoietic [20], lung [21–24], breast [24], ovarian [24, 25], colon [24], and melanoma [24]. These data demonstrate the rationale of combining SG with other clinically-relevant chemotherapeutics.

Given recent FDA approval of SG in mTNBC and accelerated approval in mUC, as well as its demonstrated clinical activity in SCLC [11], we investigated the possibility of expanding use of SG through combinations with currently utilized chemotherapeutics for these disease indications. In particular, the interaction of SG with platinum-based chemotherapeutics (carboplatin and cisplatin) in terms of *in vitro* growth inhibitory effects, cellular responses, and *in vivo* antitumor activity was examined. The results suggest that SG combined with these chemotherapeutics produce an additive growth-inhibitory effect at the low concentrations studied herein (i.e., IC_10_ to IC_30_ SG and chemotherapy concentrations), and that cells are shifted towards pro-apoptotic protein biomarker expression. Importantly, these data demonstrate significantly greater antitumor effects of SG plus carboplatin or cisplatin in tumor-bearing mice than monotherapies, and that they were well tolerated by the animals. Based on these results, SG plus platinum-based chemotherapeutics merit clinical investigation.

## RESULTS

### Trop-2 surface expression on human UBC and SCLC tumor lines

Trop-2 cell-surface expression was reported previously in various human tumor lines, including breast (TNBC, HER2^+^, and HR^+^), lung (NSCLC and squamous cell), gastric, pancreatic, and colon [26]. In addition to those already tested, several Trop-2-positive human UBC cell lines together with the SCLC tumor line DMS 53, were likewise analyzed for surface Trop-2 expression levels (Supplementary Table 1). Among UBC cell lines, surface Trop-2 expression ranged from very low in UM-UC-3 cells (2,198 ± 921 Trop-2 molecules per cell) to high in RT4 (354,641 ± 36,904 molecules per cell). In the SCLC tumor line, DMS 53, expression levels were moderate (43,620 ± 4,557 molecules per cell), being higher than those we reported for the SK-MES-1 squamous cell lung line (~29,000 molecules per cell) but lower than those reported for the Calu-3 NSCLC tumor line (~128,000 molecules per cell), both of which are sensitive to SG therapy [3, 26]. These results are consistent with past studies showing that Trop-2 expression levels within a given tumor-cell type could range from negative to high positive [26].

### *In vitro* growth inhibition of SG combined with platinum-based chemotherapeutics in TNBC, UBC, and SCLC tumor-cell types

**Figure 1:**
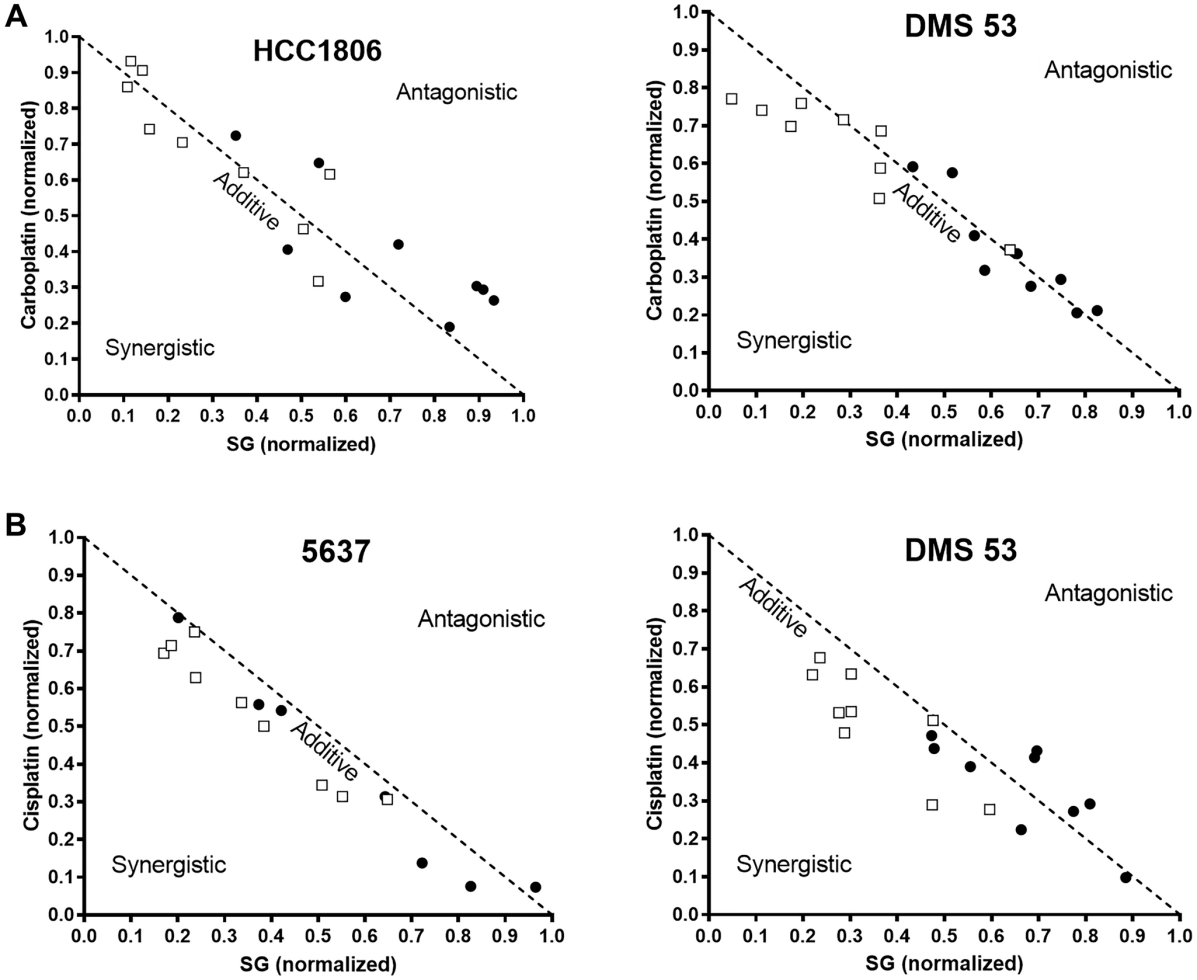
Isobolograms demonstrating changes in the *in vitro* growth-inhibitory effects of SG and platinum-based chemotherapeutics when combined in TNBC, UBC, and SCLC tumor lines. Cells were co-cultured with SG plus carboplatin or cisplatin as described in Materials and Methods. After 96 h, changes in growth inhibition were determined for SG or a given chemotherapeutic when each was incubated with a constant amount of drug (i.e., SG dose-response in constant amounts of a chemotherapeutic and *vice versa*). Isobolograms were graphed on data normalized to IC_50_-values for each individual drug (i.e., SG or chemotherapeutic). SG was combined with (**A**) carboplatin or (**B**) cisplatin. Each assay was performed at least three times for each condition (i.e., 3 assays of SG plus constant chemotherapeutic and 3 assays of chemotherapeutic in constant SG). (□) Effect on IC_50_ of the chemotherapeutic when incubated with a constant amount of SG. (●) Effect on IC_50_ of SG when incubated with constant amounts of a chemotherapeutic. Dotted line indicates additive effect of the combination. Area below and above the dotted line represent synergistic and antagonistic interactions, respectively.

Assessments were made to determine whether combinations of SG plus carboplatin or cisplatin produced synergy, additivity, or antagonism in TNBC (HCC1806), UBC (5637), and SCLC (DMS 53) cell lines (Figure 1). Carboplatin was the least cytotoxic of the agents tested with IC_50_-values greater than 3 orders of magnitude higher than SG in both DMS 53 and HCC1806 (Table 1). Isobolograms for SG plus carboplatin indicate an additive effect (Figure 1A). Calculated combination index (CI) values show a trend from antagonism at very low concentrations (IC_10_) towards synergy as the concentrations increase (Table 1). Likewise, with cisplatin, IC_50_-values were greater than 2 orders of magnitude higher when compared to SG in the 5637 and DMS 53 cell lines. When combined, SG plus cisplatin also resulted in additive growth-inhibitory effects (Figure 1B) with CI-values trending towards synergy at higher concentrations.

**Table 1:** Combination index values for SG plus carboplatin or cisplatin in various cell lines

Cell Line	Chemotherapeutic	Chemotherapeutic IC_50_-value (nM) (mean ± s.d.)	SG^a^ IC_50_-value (nM) (mean ± s.d.)	C.I.-values^b^		
				SG + Chemotherapeutic^c^ IC_10_ IC_20_ IC_30_		
DMS 53	Carboplatin	7650 ± 1160	5.61 ± 0.56	1.40	1.20	0.85
	Cisplatin	1240 ± 360		1.35	0.99	n.d.
HCC1806	Carboplatin	6790 ± 910	1.40 ± 0.33	1.69	1.19	1.01
5637	Cisplatin	340 ± 20	2.36 ± 0.22	1.68	1.17	0.72

^a^SG IC_50_-values shown in terms of SN-38 equivalents. ^b^C.I.-values = Combination Index values determined as described in Materials and Methods. (Antagonistic, C.I. >1.0; Additive, C.I. = 1.0; Synergistic C.I. <1.0). ^c^IC_10_, IC_20_, and IC_30_ = CI values determined when SG or chemotherapeutic used at concentrations that caused 10%, 20%, or 30% growth inhibition when used alone, respectively. Abbreviations: s.d.: standard deviation; n.d.: Not Determined.

### Pro-apoptotic signaling events in cells exposed to SG and carboplatin or cisplatin

**Figure 2:**
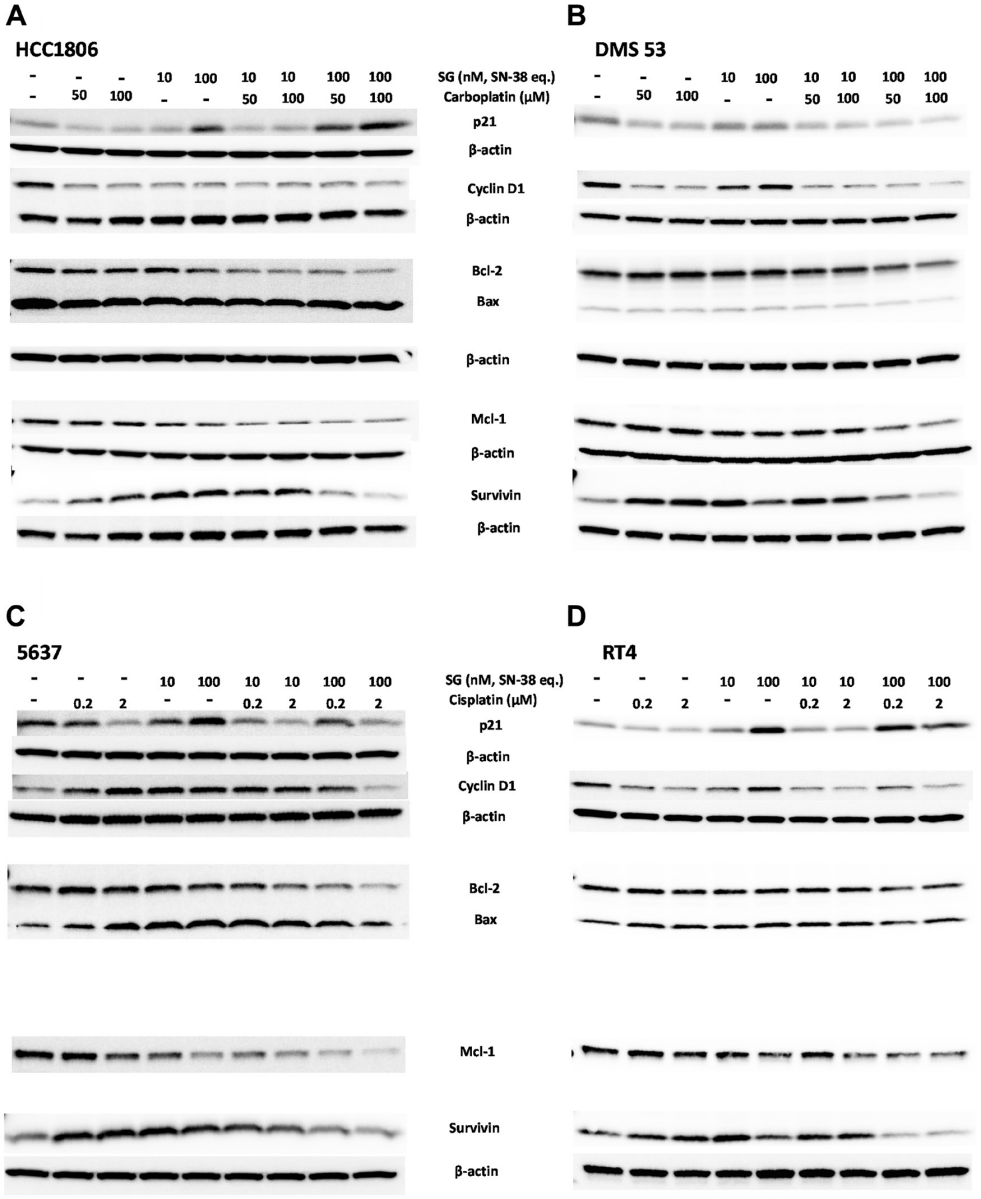
Immunoblot assessment of effects on cell-cycle, pro- and anti-apoptosis signaling events mediated by SG plus platinum-based chemotherapeutics in human TNBC, SCLC, and UBC tumor-lines. Cells were plated overnight in 6-well plates before the addition of SG and chemotherapeutics, either alone or in combination. SG concentrations are shown as SN-38 equivalents based on the protein concentration and DAR of the ADC. After a 24-h incubation, cells were harvested, and cell lysates resolved and transferred for immunoblot analysis as described in Materials and Methods. Bcl-2 and Bax levels were determined on the same blot. (**A**) Human HCC1806 TNBC cells and (**B**) DMS 53 SCLC cells exposed to carboplatin (50 and 100 μM) and SG (10 and 100 nM SN-38 equivalents). For HCC1806, β-actin loading control is the same for both Cyclin D1 and survivin blots due to stripping and re-probing the same blot. For clarity, it is reproduced under both blots. Likewise, for DMS 53, the loading control is same for p21 and Cyclin D1. Bcl-2/Bax and survivin also share the same loading control and for clarity, the same β-actin control is reproduced under Bcl-2/Bax and survivin blots. (**C**) 5637 and (**D**) RT4 human UBC human tumor lines incubated with cisplatin (0.2 and 2 μM) and SG (10 and 100 nM SN-38 equivalents). 5637 immunoblot was stripped and re-probed and therefore the β-actin loading control is the same for p21, Bcl-2/Bax, Mcl-1 and survivin. For clarity, the β-actin blot is reproduced under the p21 and survivin blots. Likewise, for RT4 p21 and Cyclin D1 share the same β-actin loading control. Bcl-2/Bax, Mcl-1, and survivin also share β-actin loading control. Each assay was performed under the same conditions at least twice.

In order to better assess how SG-mediated signaling events interact with carboplatin- and cisplatin-mediated signaling, immunoblots were performed on cell lysates after a 24-h exposure to each single agent compared to various combinations (Figure 2). Possible effects on cell cycle were measured as changes in p21^Waf1/Cip1^ and Cyclin D1 expression [27–30], whereas pro-apoptotic signaling was measured as changes in the Bax:Bcl-2 ratio [31]. Changes in expression of anti-apoptotic proteins, Mcl-1 and survivin, were likewise determined in the cells [32, 33].

SG was combined with either carboplatin in TNBC and SCLC tumor lines (HCC1806 and DMS 53; Figure 2A, 2B, respectively) or with cisplatin in two UBC tumor lines (5637 and RT4; Figure 2C, 2D, respectively). Previous studies demonstrated SG mediated the up-regulation of p21^Waf1/Cip1^ in pancreatic, NSCLC, and gastric carcinoma cell lines within 24 h of continuous exposure and to arrest TNBC cells in the S-phase of the cell cycle [3, 17, 26]. Consistent with these past results, SG treatment resulted in the up-regulation of p21^Waf1/Cip1^ in 3 of the 4 cell lines tested with only DMS 53 demonstrating unchanged expression relative to untreated control cells. The degree to which p21^Waf1/Cip1^ is up-regulated and down-regulated is related to the degree of DNA damage (low levels *versus* high, respectively) and is the difference between cytostatic *versus* cytotoxic drug-levels [34]. Likewise, under DNA damaging conditions, Cyclin D1 is known to be recruited to help repair the damage (i.e., up-regulated) but will decrease under conditions of severe damage [30, 35]. Further, since SG mediates cell-cycle arrest in the S-phase, and Cyclin D1 is known to be degraded as the cell progresses from G1 to S-phase, one would likewise expect that levels of Cyclin D1 would decrease as cells accumulate in the S-phase [35]. However, while this was the case in one of the tumor lines, HCC1806, the other three either showed no change (DMS 53 and RT4) or, conversely, induced an up-regulation of Cyclin D1 (5637). Monoculture with the platinum-based chemotherapeutics had little effect on p21^Waf1/Cip1^ expression levels except for the 5637 UBC cell line, in which the highest cisplatin concentration down-regulated p21^Waf1/Cip1^. Both carboplatin and cisplatin mediated the down-regulation of Cyclin D1 in all the cell lines except for 5637, in which Cyclin D1 was up-regulated in the treated cells. When combined at their respective high concentrations, SG plus carboplatin or cisplatin resulted in the down-regulation of both p21^Waf1/Cip1^ and Cyclin D1 relative to monoculture of the individual drugs. Only in HCC1806 did the combination of SG and carboplatin appear to have no effect on p21^Waf1/Cip1^ or Cyclin D1 relative to individual exposures levels. These data suggest that individual drugs are perturbing the normal cell-cycle of the cells with the greatest effect occurring when SG is combined with either carboplatin or cisplatin.

In terms of anti- and pro-apoptotic protein expression, all four cell lines tested readily expressed basal levels of the anti-apoptotic Bcl-2 protein. Pro-apoptosis Bax protein levels, however, were variable with high basal expression in HCC1806 cells to low in DMS 53. In HCC1806 cells, the level of Bax protein remained relatively unchanged whereas expression of Bcl-2 was down-regulated when SG and carboplatin were combined, resulting in an increased Bax to Bcl-2 ratio (Table 2). In contrast, for DMS 53, both were down-regulated upon co-culturing but overall, there was little change in the Bax and Bcl-2 ratios at the lower concentrations but there was a shift in favor of Bcl-2 at the higher combinations suggesting the cells were shifting to a more anti-apoptosis signaling position at the concentrations tested here. Interestingly, in 5637, both cisplatin and SG alone resulted in up-regulation of Bax while Bcl-2 levels fell. When SG was combined at the highest cisplatin concentration, the ratio of Bax to Bcl-2 was further increased to greater than 3-fold above baseline, suggesting these cells were particularly sensitive to this combination. In RT4 cells, each single agent mediated an increase in the Bax to Bcl-2 ratio, although the combination of SG and cisplatin did not substantially alter the ratio from that observed for each agent alone. Overall, these data show that combining SG with either carboplatin or cisplatin will result in suppression of anti-apoptotic Bcl-2 protein expression in concert with an increase in the expression of pro-apoptotic Bax relative to Bcl-2, thereby shifting the cells in the direction of apoptosis.

**Table 2:** Calculated Bax:Bcl-2 Immunoblot ratios in various tumor lines incubated with SG plus carboplatin or cisplatin

Cell Line	Treatment	Bax:Bcl-2 Ratio (SG + Chemotherapeutic)			
		μM	SG (nM)^b^		
			0	10	100
HCC1806	Carboplatin	0	1^a^	0.71	0.97
		50	0.91	1.33	1.37
		100	0.86	1.48	1.73
DMS 53	Carboplatin	0	1^a^	1.21	1.08
		50	1.09	1.00	0.85
		100	0.97	0.92	0.70
5637	Cisplatin	0	1^a^	2.06	2.42
		0.2	1.24	2.34	2.88
		2	1.99	3.06	3.59
RT4	Cisplatin	0	1^a^	1.33	1.52
		0.2	1.28	1.42	1.38
		2	1.34	1.39	1.55

^a^Bax:Bcl-2 background ratios (i.e., cells incubated in media alone) normalized to 1. Shaded areas indicate the ratios when the SG was combined with a given drug (carboplatin or cisplatin) at a given set of indicated concentrations. All these ratios are based on values relative to normalized background control. ^b^SG concentrations shown as SN-38 equivalents.

Finally, the cell’s ability to express Mcl-1 and survivin apoptosis-inhibitory proteins was determined for SG and carboplatin/cisplatin combinations. In all four cell lines, Mcl-1 was constitutively expressed at its highest expression level in the untreated cells. These levels fell to their lowest point when the cells were incubated with both SG and either carboplatin or cisplatin. Unlike Mcl-1, survivin levels were up-regulated when cells were exposed to each single chemotherapeutic agent or with SG. When cells were incubated with SG at 100 nM and either carboplatin or cisplatin, survivin expression fell to baseline levels or lower. These data demonstrate that when SG and carboplatin or cisplatin are combined, a cell’s anti-apoptosis response is hindered by way of inhibiting expression of both Mcl-1 and survivin. Taken together, all these data suggest that when combined, SG plus platinum-based chemotherapeutics alter normal cell-cycle progression and activate those signaling pathways that favor a more pro-apoptotic condition.

### Efficacy of SG plus cisplatin or carboplatin in mice bearing human TNBC, SCLC, and UBC tumor xenografts

**Figure 3:**
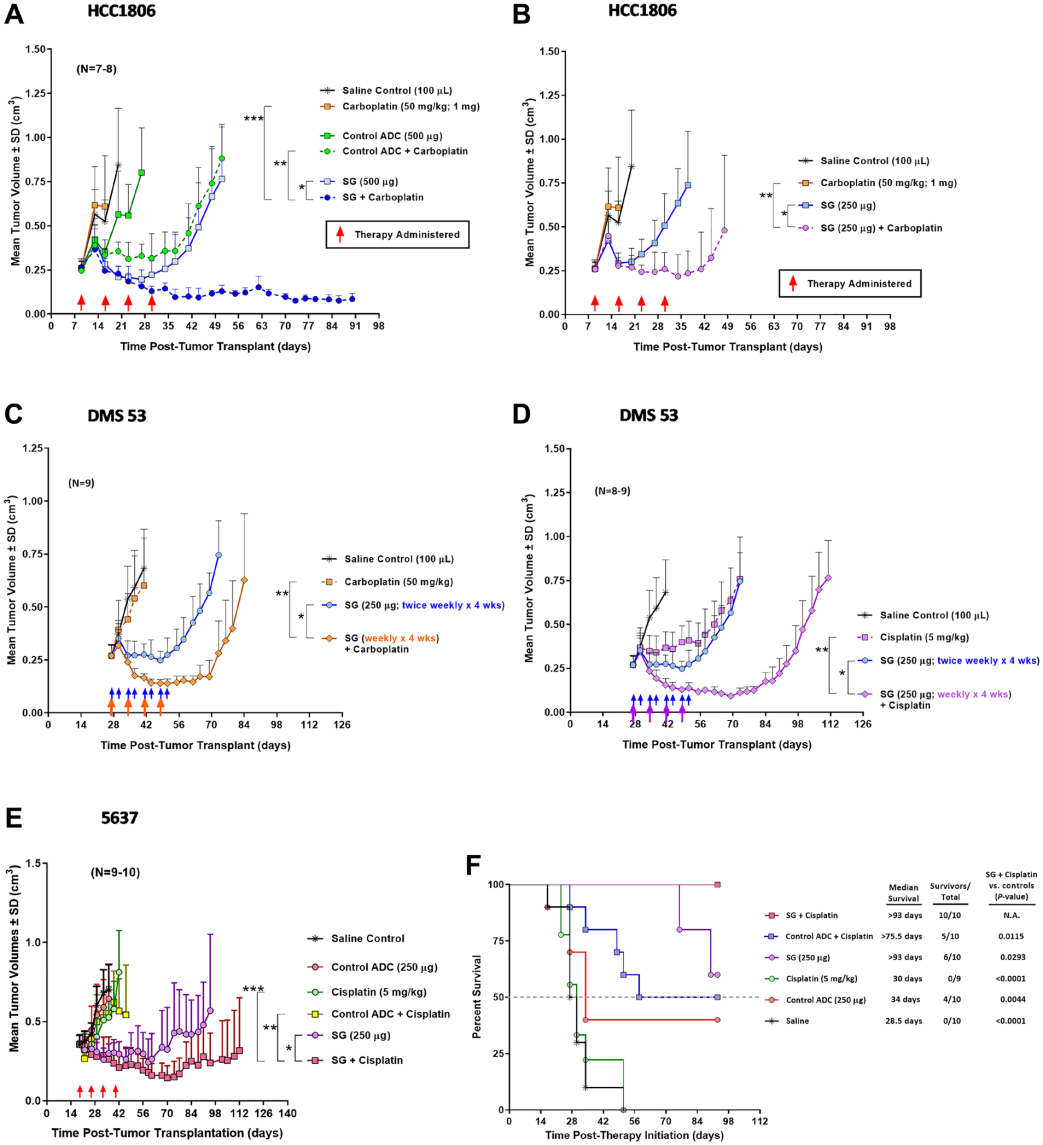
*In vivo* efficacy of SG combined with carboplatin or cisplatin in mice bearing human TNBC, SCLC, or UBC xenografts. Animals were set up with the various tumor xenografts as described in Materials and Methods. Mice were treated with SG i.v., carboplatin i.p., cisplatin i.p., control ADC i.v. (h679-CL2A-SN-38), or combinations at the indicated doses. Brackets in all the figures represent AUC *P*-values between the two indicated treatment comparisons for a given set of groups. (**A**, **B**) Tumor growth curves of mice bearing HCC1806 human TNBC xenografts treated with the combination of SG plus carboplatin. Both SG and carboplatin were administered once weekly for four weeks (red arrows). (A) Mice administered 500 μg SG alone or in combination with carboplatin. (^*^ *P* = 0.0006, ^**^ *P* < 0.0001, and ^***^ *P* = 0.0062). (B) Mice administered 250 μg SG alone or in combination with carboplatin. These mice were in the same study as in (A) and thus share the same control groups. (^*^ *P* = 0.0047 and ^**^ *P* = 0.0363). (**C**) DMS 53 tumor growth curves for mice treated with SG plus carboplatin. SG alone groups received twice weekly injections (blue arrows) while in the combination group, SG was administered on the same schedule as the carboplatin (i.e., weekly x 4 wks; orange arrows). (^*^ *P* < 0.0001 and ^**^ *P* = 0.0017). (**D**) Likewise, DMS 53 tumor-bearing mice treated with SG plus cisplatin weekly for four weeks (purple arrows). These mice were in the same study as in (C) and therefore share the same saline and SG monotherapy control groups. (^*^ *P* < 0.0001 and ^**^ *P* < 0.0001). (**E**) Mice bearing 5637 human UBC tumors and treated with the combination of SG plus cisplatin weekly for four weeks (red arrows). (^*^ *P* = 0.0362, ^**^ *P* = 0.0007, and ^***^ *P* = 0.0030). (**F**) Survival curves for those mice bearing the 5637 tumors and treated with SG plus cisplatin. Dotted grey line denotes the 50% survival threshold. Log-rank analysis was utilized to calculate the *P*-values. Abbreviations: N.A.: Not Applicable. SD: Standard Deviation.

To determine if the observations made *in vitro* suggesting enhanced growth inhibitory effects and pro-apoptosis signaling events of SG plus carboplatin or cisplatin translate into improved efficacy *in vivo*, several studies were performed to test these combinations in murine models of human disease (Figure 3). SG, carboplatin, and cisplatin dose/schedules were chosen to produce a minimal antitumor effect when used alone to better gauge the effect when combined in tumor-bearing animals. Mice bearing HCC1806 tumors treated with carboplatin exhibited no antitumor effects while animals treated with the higher of two SG doses (500 μg) demonstrated significant tumor regressions compared to carboplatin and a non-specific control ADC (Figure 3A, *P* < 0.0223, AUC). Importantly, when combined, SG and carboplatin provided significant and long-lasting antitumor effects compared to all controls, including SG alone and non-specific ADC plus carboplatin (Supplementary Table 2; *P* ≤ 0.0062, AUC). While SG monotherapy produced significant tumor regressions during the time animals were being treated in this aggressive tumor model (median time to nadir = 18 days post-therapy initiation), animals began to show disease progression at a median of 7 days after the end of therapy. Conversely, mice treated with the combination of SG and carboplatin exhibited tumors significantly smaller on the day the study ended compared to when therapy began 81 days earlier (TV = 0.085 ± 0.032 cm^3^ vs. 0.266 ± 0.035 cm^3^, day 81 post-therapy initiation vs. day 0, respectively; *P* < 0.0001). Even when the SG dose was reduced by 50% (250 μg), combination therapy produced significant antitumor effects when compared to SG and carboplatin monotherapy (Figure 3B; Supplementary Table 2; *P*≤0.0363, AUC). Interestingly, the combination of carboplatin plus a non-specific control ADC (500 μg) produced significant antitumor effects compared to monotherapy with either agent (*P* < 0.0228, AUC). This appears to be related to the overall sensitivity of these tumors to these agents coupled with the hydrolysable CL2A linker and release of the SN-38 providing bystander killing of non-targeted tumor cells. This combination of SG plus carboplatin was well tolerated, as determined by changes in body weight with no treatment-related loss of any of the animals (Supplementary Figure 1A).

Mice bearing human SCLC tumor xenografts (DMS 53) were likewise treated with a combination of SG plus carboplatin on a weekly basis for four weeks (Figure 3C). Monotherapy of SG produced significant antitumor effects in the mice when compared to both carboplatin monotherapy and saline control (*P* < 0.0095, AUC). When SG was administered with carboplatin, tumors exhibited significantly greater tumor regressions compared to SG monotherapy (Supplementary Table 2; *P* = 0.0001, AUC). This improved efficacy occurred even though the SG dosing was cut by 50% in the combination group compared to SG monotherapy (i.e., SG administered weekly × 4 weeks in the combination group vs. twice weekly × 4 weeks in the SG monotherapy group). Animals treated with the combination resulted in an 89% PR and 11% with SD compared to 100% SD in the SG monotherapy group as best response (Table 3). None of the animals treated with carboplatin alone exhibited any antitumor effects. Further, the SG plus carboplatin combination mediated significantly greater TTP when compared to all control treatment groups (Table 3; *P* ≤ 0.0002).

**Table 3:** Time to tumor progression in DMS 53 tumor-bearing mice treated with the combination of SG plus cisplatin or carboplatin

Treatment	TTP (days) (mean ± s.d.)	CR%^a^	PR %^b^	SD %^c^	ORR %^d^	TTP Comparisons	
						SG + Cisplatin vs. Controls (*P*-value)	SG + Carboplatin vs. Controls (*P*-value)
SG + Cisplatin	47.3 ± 4.5	0	100	0	100	n.a.	n.a.
SG + Carboplatin	41.7 ± 8.2	0	89	11	89	0.1093	n.a.
SG alone^e^	26.4 ± 4.7	0	0	100	0	<0.0001	0.0002
Cisplatin alone	10.6 ± 10.2	0	0	44	0	<0.0001	<0.0001
Carboplatin alone	4.3 ± 2.0	0	0	0	0	<0.0001	<0.0001
Saline^e^	3.9 ± 1.8	0	0	0	0	<0.0001	<0.0001

TTP, Time post-therapy initiation when TV progressed to >20% of its nadir. ^a^CR%, percent of mice that exhibited a complete response as best response (i.e., tumors regressed 100%). ^b^PR%, percent of mice that exhibited a partial response as best response (i.e., tumors regressed ≥30%). ^c^SD%, percent of mice that exhibited stable disease as best response (i.e., tumors stayed between 70% to 120% of initial volume). ^d^ORR %, overall response rate (CR + PR). ^e^Both the SG plus cisplatin and SG plus carboplatin study was performed as a single experiment and therefore share saline control and SG alone control groups. Abbreviations: s.d.: standard deviation; n.a.: Not applicable.

Unlike carboplatin, DMS 53 tumor-bearing animals treated with cisplatin monotherapy demonstrated significant antitumor effects compared to saline control (Figure 3D; *P* = 0.0035, AUC). When combined, SG plus cisplatin resulted in significant tumor regressions compared to mice treated with either SG or cisplatin monotherapies (Supplementary Table 2; *P* < 0.0001, AUC). Both SG and cisplatin monotherapy groups lost their first animals due to disease progression 46 days after initiation of therapy. On this day, mean tumor volumes in the SG plus cisplatin treatment group were significantly smaller than those in either monotherapy group (Supplementary Table 2; TV = 0.112 ± 0.026 cm^3^ vs. 0.745 ± 0.162 and 0.758 ± 0.244 cm^3^, combination vs. SG and cisplatin monotherapies, respectively; *P* < 0.0001). Additionally, combining SG with cisplatin resulted in a 100% PR rate compared to only 44% SD for the cisplatin monotherapy group (Table 3). TTP was significantly longer for the combination compared to either monotherapy (*P* < 0.0001). While TTPs were similar for both SG plus cisplatin and SG plus carboplatin combinations, the SG plus cisplatin therapy produced a greater degree of tumor regressions (*P* = 0.0024, AUC). On the day the first animal was lost due to disease progression in the SG plus carboplatin group (57 days post-therapy initiation), mice in the SG plus cisplatin group had significantly smaller tumors than those in the carboplatin combination group (0.176 ± 0.05 cm^3^ vs. 0.628 ± 0.312 cm^3^, respectively; *P* = 0.0023).

Mice bearing the DMS 53 tumors exhibited cachexia as evidenced by a greater than 15% loss in weight as the tumors progressed in all the treatment groups during the early stages of therapy, including saline control. Not only were the combinations of SG with either carboplatin (Supplementary Figure 2A) or cisplatin (Supplementary Figure 2B) well tolerated by the mice, these were the only groups that demonstrated gain in weight as the tumors regressed during treatment and after the end of therapy. Only when the tumors began to progress in the combination groups several weeks after therapy was ended did the mice again show signs of cachexia. These data demonstrate that not only were the SG/carboplatin and SG/cisplatin combinations well tolerated by the animals, but their ability to cause significant and prolonged tumor regressions also helped to reverse the cachexia associated with this SCLC tumor model.

Lastly, mice bearing human 5637 UBC tumor xenografts were treated with SG and cisplatin weekly for four weeks (Figure 3E). Compared to all other control groups, including a non-specific ADC plus cisplatin, the SG plus cisplatin combination produced significantly greater antitumor effects (Supplementary Table 2; *P* ≤ 0.0362, AUC). This tumor growth-inhibitory effect translated into a significant survival benefit (Figure 3F; *P* ≤ 0.0293, log-rank). Combining SG plus cisplatin was well tolerated with no treatment-related loss of any of the animals (Supplementary Figure 1B).

## DISCUSSION

Monotherapy with SG proved to be clinically efficacious in several different types of solid tumors, including SCLC, mTNBC, mHR^+^/HER2^-^ BC, and mUC, with regular FDA approval in mTNBC, mHR^+^/HER2^-^ BC, and accelerated approval for mUC [7–13]. Many current treatment approaches to these, and other solid tumor disease indications, often rely on combining therapies, especially with non-overlapping toxicities, to improve therapeutic outcomes [16]. We previously demonstrated preclinically the utility of combining SG with PARPi’s which is being further investigated clinically [17–19]. In an active clinical trial in which mTNBC patients are being treated with SG plus talazoparib, early indications of clinical activity were demonstrated through the accumulation of a greater amount of dsDNA breaks in on-treatment specimens when compared with paired pre-treatment specimens. Further, this combination, using staggered dosing, was found to be well-tolerated without dose-limiting toxicities, thereby showing the effectiveness of this combination [19]. Herein, we sought to examine the effect of combining SG therapy with two other common types of chemotherapeutics, namely carboplatin and cisplatin, and to determine if such an approach may warrant further clinical development.

Studies that examined co-culturing of cisplatin with topotecan or SN-38 showed the interaction to be synergistic in several different tumor lines including lung (SCLC and NSCLC) and breast [21–24]. Co-culturing of SG with either cisplatin or carboplatin produced additive growth inhibitory effects in the TNBC, UBC, and SCLC tumor lines studied here. It should be noted that the combinations tested were at low concentrations (i.e., IC_10_-IC_30_ range) while others found that synergy was achieved at higher concentrations (e.g., IC_50_ and higher), but when the concentrations were lowered (e.g., IC_10_ or IC_20_), the interaction become additive and, in some cases, antagonistic [24]. It may be that at these lower concentrations, the amount of DNA damage is such that the cell is able to trigger proficient DNA repair pathways that blunt the cytotoxic effect of these agents. Such DNA repair activation may also account for antagonism at these lower concentrations. In a TNBC cell line resistant to the antitumor effects of SG due to activation of the homologous DNA repair pathway (HRR), significant tumor regressions could be achieved if the tumor line was transfected to express greater amounts of surface Trop-2 [36]. Given that mice bearing these tumors were still resistant to irinotecan administrations, it was hypothesized that by increasing the levels of Trop-2 in the tumor cells, a greater amount of SN-38 was delivered by SG resulting in pushing the cells past a DNA damage threshold that even its proficient HRR pathway was unable to overcome resulting in triggering apoptosis and cell death. In cell lines with either acquired cisplatin resistance or intrinsically resistant, when combined with SN-38 there was an increase in intracellular platinum levels [25]. However, the mechanisms by which the combination of a platinum-based chemotherapeutic and a TOP1 inhibitor affect cells may be cell-line dependent. In an ovarian tumor line and its cisplatin resistant clone, the combination of topotecan and cisplatin increased the amount of interstrand cross-links (ICL) whereas in a SCLC tumor line, cisplatin plus SN-38 did not result in an increase in ICLs [22, 24]. Future experimentation will seek to determine whether SG will likewise mediate increases in ICLs as well as produce increased intracellular levels of platinum in cisplatin and carboplatin resistant tumor cells.

An important observation previously reported was that pre-treatment of tumor cells with cisplatin prior to the addition of SN-38 produced an even greater, synergistic growth-inhibitory effect [24]. As such, the effect of sequencing of cisplatin or carboplatin with SG may prove important in terms of both tolerability and efficacy as noted below when SG was combined with PARP inhibitors [18, 19]. Clinically, cisplatin has a monoexponential plasma clearance with a half-life of ~0.5 h [37]. Carboplatin has a biphasic clearance from the plasma with a 1.1–2.0 h alpha-phase (t½_α_) and 2.6–5.9 h beta-phase half-life with an overall plasma mean residence time of 3.5 h [38]. This plasma clearance of both drugs is similar in mice with a t½_α_ of ~0.5 h with the bulk of drug being cleared renally during the first 24 h post-infusion [39]. Conversely, SG has a much longer half-life with accumulation in the tumor over a 72-hour period-of-time [3, 5]. Given these PK differences, *in vivo* co-administration was in a sense pre-exposing the tumor cells to the cisplatin or carboplatin prior to SG. When tested in tumor-bearing animals, both cisplatin and carboplatin combinations with SG did produce significant antitumor effects across all three disease models (TNBC, UBC, and SCLC) when co-injected, with no observable toxicities. While modest antitumor effects were observed when the non-specific control ADC was combined with these platinum-based drugs, it is likely attributed to extracellular release of drug and the overall sensitivity of a specific tumor to the combination [6]. Recent studies demonstrate that SG produces significantly greater DNA damage and SN-38 payload diffusion throughout a tumor (i.e., bystander effect) than a non-specific ADC due to SG’s rapid internalization and efficient payload release mediated by its uniquely hydrolysable linker [6]. Moreover, co-administration of these small drug molecules with SG is in effect treating the tumor cells with the smaller drug first, which is followed by delivering an effective dose of SN-38 days later as SG accretes in the tumor.

Both SG and platinum-based chemotherapeutics trigger cell apoptosis via the intrinsic apoptosis pathway through accumulated DNA damage [26, 40–47]. In terms of possible overlapping toxicities, main toxicities associated with these platinum-based drugs are hematologic, nephrotoxic, and gastrointestinal (GI) with only the effect on bone marrow (i.e., neutropenia) and GI being shared with SG, which can be treated and managed in patients [7, 14, 37, 38]. Previous non-clinical studies examining the effect of combining SG with PARP inhibitors (olaparib, rucaparib, and talazoparib) demonstrated no observable toxicities in mice [17]. However, when SG was co-administered with rucaparib clinically in a phase Ib trial (NCT03992131), all the patients experienced dose-limiting toxicities (DLTs) which included neutropenia. These DLTs required a combination of treatment interruptions, dose reductions, or granulocyte colony stimulating factor administration. No optimal recommended phase II dose was established in this combination study. It was recommended that future trials examine the use of intermittent dosing of SG and PARP inhibitors to reduce myelosuppression and optimize antitumor efficacy [18]. In a similar phase Ib clinical trial (NCT04039230), SG was combined with a different PARP inhibitor (talazoparib) in patients with mTNBC. A staggered dosing schedule was employed together with supportive therapy resulting in a relatively well-tolerated therapy without DLTs that produced promising clinical activity [19]. A recommended phase II dose from this study was determined to be sequential SG (10 mg/kg on days 1 and 8) with talazoparib (1 mg on days 15–21), every 21 days. Using a similar staggered dosing approach combining SG with these platinum-based chemotherapeutics may likewise produce improved clinical safety profiles without sacrificing efficacy.

SG mediates the induction of p21^Waf1/Cip1^ which in-turn results in cells accumulating in the S-phase of the cell cycle [1, 3, 17]. Likewise, both cisplatin and carboplatin arrest cells in the G1/S-phase of the cell cycle [48, 49]. Further, p21^Waf1/Cip1^ is up-regulated upon low levels of DNA damage and down-regulated during times of high DNA damage. This is thought to be related to the difference between cytostatic *versus* cytotoxic drug-levels [34]. When combined with SG, the addition of either carboplatin or cisplatin inhibited SG-mediated p21^Waf1/Cip1^ up-regulation in all the cell lines except in the HCC1806 TNBC tumor line, indicating that for those cell lines affected, the drug combinations were pushing cells into a more cytotoxic state. It is possible that for the one cell line whose p21^Waf1/Cip1^ levels remained elevated when SG was combined with carboplatin (HCC1806), the doses used only damaged the DNA enough to stall the cells in the S-phase. Another cell-cycle regulating protein, Cyclin D1, is typically degraded as cells enter the S-phase of the cell cycle but is also known to be recruited by the DNA HRR pathway in the cell’s effort to repair damage, but it too will decrease if DNA damage is significant [30, 35]. Only in 5637 UBC cells did we observe up-regulation of Cyclin D1 in response to monoculture with SG or cisplatin. This particular tumor line has a robust HRR response to DNA damage and may be recruiting Cyclin D1, which is consistent with these results [50]. Importantly, in all the cell lines, combinations of SG with platinum-containing chemotherapeutics resulted in down-regulation of Cyclin D1. Only in the HCC1806 TNBC tumor line did we observe consistent down-regulation of Cyclin D1 for all culture conditions (i.e., monoculture and combinations). This is not surprising since it is the only one of the four cell lines tested to have defective HRR pathways, and therefore would have no need to recruit this protein to repair damaged DNA [36].

Cell death mediated by the SN-38 drug-payload of SG via the intrinsic apoptosis pathway begins with release of cytochrome C from the mitochondria and subsequent activation of a caspase cascade beginning with cleavage of caspase 9 into its active form [26, 44]. Bax likewise promotes release of cytochrome C whereas both Bcl-2 and the Bcl-2-family protein, Mcl-1, block this release and thus impede activation of the caspase cascade [32]. The main effect of these SG combinations was not to change the expression of Bax, but rather to decrease the level of Bcl-2 expression, thus shifting the ratio in favor of Bax. Likewise, Mcl-1 levels fell to the greatest degree upon exposure to the combinations of SG plus cisplatin or carboplatin. Another anti-apoptosis protein, survivin, impacts activation of the intrinsic apoptosis pathway by blocking activation of caspase 9. In fact, survivin mRNA is one of the more frequently upregulated mRNAs in human cancer transcriptomes [33]. While monotreatment of cell lines resulted in up-regulation of survivin, when combined, levels remained at or below constitutive levels. Altogether, combinations of SG and platinum-based drugs appear to mediate cellular conditions that promote cell-cycle arrest and pro-apoptosis signaling events in these TNBC, SCLC, and UBC tumor lines.

These studies sought to make initial assessments of potential mechanisms that may play a role in enhancing pro-apoptotic signaling events in the cells when SG was combined with these platinum-based chemotherapeutics. There are many other signaling proteins that can also be considered in future studies including those associated with cell-cycle (e.g., phosphorylation of other cyclin dependent kinases) [28], apoptosis (e.g., caspase activation) [44], and DNA damage (e.g., γ-H2A.X) [51]. Further, the effect of long-term exposure to these platinum-based chemotherapeutics plus SG in terms of resistance have not been studied here. Future studies will make use of patient-derived xenografts for each indication, including those obtained from carboplatin and cisplatin refractory patients. One possible mechanism of acquired resistance may be changes in expression of Trop-2. Recent evidence found that one mechanism of SG resistance is a mutational change in the Trop-2 protein *TACSTD2/TROP2* with a T256R mutation resulting decreased plasma membrane localization compared to the wild-type protein with an 80% loss in SG binding [52]. Studies will examine this and other biomarkers associated with each of the individual disease indications to better ascertain their translational significance in the clinical setting.

In conclusion, these results support the rationale and potential for favorable clinical outcomes of combining SG therapy with platinum-based chemotherapeutics in solid tumors. Additionally, the possibility of other combinations of SG with inhibitors against anti-apoptosis proteins (i.e., Bcl-2, Mcl-1, survivin) should also be explored given the role these proteins play in blocking the intrinsic apoptosis signaling pathway typically activated by the SN-38 payload of SG. Moreover, as SG monotherapy has shown efficacy with manageable toxicities in regulatory-approved mTNBC and mUBC indications, combining SG with currently approved platinum-based drugs for these and other SG-responsive tumors (e.g., SCLC), may enhance clinical benefit without compromising tolerability.

## MATERIALS AND METHODS

### Cell lines, antibody-drug conjugates, antibodies, and chemotherapeutics

All cell lines were purchased from American Type Culture Collection (ATCC; Manassas, VA) and maintained according to ATCC recommendations. Any cell line with an unknown passage number was authenticated by short tandem-repeat assay by the ATCC. SG, control ADC (h679-CL2A-SN-38 (humanized anti-histamine-succinyl-glycine antibody conjugated with SN-38 via the CL2A linker)), and hRS7 IgG were prepared at Immunomedics, Inc. (Morris Plains, NJ). For *in vitro* assays and immunoblots, SG is expressed in terms of SN-38 equivalents which is based on protein concentration and DAR. For example, based on a mean DAR of 7, a concentration of 14.3 nM SG is equivalent to 100 nM SN-38. Carboplatin (Sagent Pharmaceuticals Inc., Shaumburg, IL) and Cisplatin (Teva Pharmaceuticals USA, Inc., North Wales, PA) were purchased and further diluted into working solutions in sterile saline (0.9% NaCl, Injection, USP; Hospira Inc.).

### Assessment of Trop-2 expression on cell lines

Expression of Trop-2 on the cell surface is based on flow cytometry making use of QuantiBRITE PE beads (BD Cat. No. 340495) and a PE-conjugated anti-Trop-2 antibody (eBiosciences, Cat. No. 12-6024), as described previously [26] and presented in the Supplementary Materials.

### *In vitro* combination cytotoxicity assays

*In vitro* cytotoxicity was determined using the 3-(4,5-dimethylthiazol-2-yl)-5-(3-carboxymethoxyphenyl)-2-(4-sulfophenyl)-2H-tetrazolium dye reduction assay (MTS dye reduction assay; Promega, Madison, WI). Drug combination assays were performed as described previously [17] and further presented in the Supplementary Materials. Dose-response curves for each agent alone were first tested to determine single agent IC_10_-, IC_20_-, or IC_30_–values after 96-h incubation before combinations of SG plus either cisplatin or carboplatin were tested for additivity, synergy, or antagonism.

### Immunoblot assessment of SG- and chemotherapy-mediated cell signaling *in vitro*

Details of these immunoblot studies are given in the Supplementary Materials. Briefly, cells were plated overnight in 6-well plates. The following day, SG, a chemotherapeutic (carboplatin or cisplatin), or the combination of SG and a chemotherapeutic were added to appropriate wells for 24 h. Concentrations for each agent is shown in the figure. Cells were harvested, lysed and a total of 20 μg protein was resolved in 4–12% Bis-Tris NuPAGE gels (Thermo Fisher Scientific; Cat. No. NP0322) and transferred to polyvinylidene difluoride (PVDF) membranes. Membranes were probed overnight at 4°C with primary antibody, followed by 1 h incubation at room temperature with secondary antibody.

### *In vivo* therapeutic studies

All animal studies were approved by Montclair State University Institutional Animal Care and Use Committee (Montclair, NJ). Details of tumor models are described in the Supplementary Materials. Tumor volume (TV) was determined by measurements in two dimensions using calipers, with volumes defined as: *L* × *w*^2^/2, where *L* is the longest dimension of the tumor and *w* the shortest. Mice were randomized into treatment groups and therapy began when TVs were approximately 0.3 cm^3^. Treatment regimens, dosages, and number of animals in each experiment are described in the *Results* and in the Figure Legends.

Mice were deemed to have succumbed to disease progression and euthanized once tumors grew to > 1.0 cm^3^ in size. A partial response (PR) is defined as shrinking the tumor >30% from initial size. Stable disease (SD) is when the tumor volume remains between 70% and 120% of initial size. Time-to-tumor progression (TTP) was determined as time post-therapy initiation when TV increased more than 20% from its nadir.

Toxicity was assessed in the animals based on body mass changes. Animals that lost more than 15% of starting weight were monitored daily and euthanized if they did not gain back weight within two days. The only exception was in mice bearing DMS 53 tumors where tumor-induced cachexia occurred. These animals were monitored throughout the study and euthanized only when TV exceeded 1.0 cm^3^ which typically corresponded to weight loss greater than 20%.

### Statistical analysis

Grubb’s critical-Z test was performed on tumor-progression data for the treatment and control groups, with *P* ≤ 0.05 for any mouse deemed an outlier. Such mice were removed from further statistical analysis. No more than one mouse was ever removed from a group based on this statistical test. Statistical analysis of tumor growth was based on area-under-the-curve (AUC). Profiles of individual tumor growth were obtained through linear-curve modeling. An F-test was employed to determine equality of variance between groups prior to statistical analysis of growth curves. A two-tailed *t*-test was used to assess statistical significance between the various treatment groups and controls, except for the saline/untreated controls, where a one-tailed *t*-test was used in the comparison. Survival studies were analyzed using Kaplan-Meier plots (log-rank analysis), using Prism GraphPad Software package (v6.05; Advanced Graphics Software, Inc.; Encinitas, CA). Significance was set at *P* ≤ 0.05.

## SUPPLEMENTARY MATERIALS


