# Supplementary material for: Sacituzumab govitecan plus platinum-based chemotherapy mediates significant antitumor effects in triple-negative breast, urinary bladder, and small-cell lung carcinomas
Source: Oncotarget. 2024 Feb 22;15:144–58. doi: 10.18632/oncotarget.28559 (PMC10883684; doi:10.18632/oncotarget.28559)
Supplement: Supplementary file 1 [file oncotarget-15-28559-s001.pdf]

Sacituzumab govitecan plus platinum-based chemotherapy mediates significant antitumor effects in triple-negative breast, urinary bladder, and small-cell lung carcinomas

SUPPLEMENTARY MATERIALS

Assessment of Trop-2 expression on cell lines

Expression of Trop-2 on the cell surface is based on flow cytometry. Briefly, cells were harvested with Accutase Cell Detachment Solution (Becton Dickinson (BD), Franklin Lakes, NJ; Cat. No. 561527) and assayed for Trop-2 expression using QuantiBRITE PE beads (BD Cat. No. 340495) and a PE-conjugated anti-Trop-2 antibody (eBiosciences, Cat. No. 12-6024) following the manufactures' instructions. Data were acquired on a FACSCalibur Flow Cytometer (BD) with CellQuest Pro software. Staining was analyzed with Flowjo software (Tree Star, Ashland OR).

In vitro combination cytotoxicity assays

In vitro cytotoxicity was determined using the 3-(4,5-dimethylthiazol-2-yl)-5-(3-carboxymethoxyphenyl)-2-(4-sulfophenyl)-2H-tetrazolium dye reduction assay (MTS dye reduction assay; Promega, Madison, WI). Briefly, cells were plated into 96-well clear, flat-bottomed plates overnight. Dose-response curves for each agent alone were first tested to determine single agent IC_{10}-, IC_{20}-, or IC_{30}–values after 96-h incubation. In combination assays, one agent (e.g., SG) is tested on a given cell line across a range of concentrations (i.e., dose-response curves) either alone or in the presence of a constant amount (IC_{10}-, IC_{20}-, or IC_{30}–concentration) of the second agent (e.g., carboplatin). This is then reversed in which the chemotherapeutic was plated across a concentration range either alone or with a constant amount of SG (i.e., IC_{10}-, IC_{20}-, or IC_{30}–concentration). Growth inhibition was measured as a percent of growth relative to untreated cells. Dose/response curves were generated from the mean of triplicate determinations, and IC_{50}-values were calculated using Prism GraphPad Software package (v6.05; Advanced Graphics Software, Inc.; Encinitas, CA). For each condition, the IC_{50}-value was determined from these data. These IC_{50}-vlaues are then normalized and plotted as isobolograms or used to calculate combinatorial Index (CI) numbers.

CI were calculated using the following formula:

$$CI = Da/Dxa + Db/Dxb \ [1],$$

where,

$Da = IC_{50}$ of SG when used in combination with a constant amount of a given chemotherapeutic (IC_{10}, IC_{20}, or IC_{30} concentration);

$Db = IC_{50}$ of chemotherapeutic when used in combination with a constant amount of SG (IC_{10}, IC_{20}, or IC_{30} concentration);

$Dxa = IC_{50}$ of SG when used alone; and

$Dxb = IC_{50}$ of chemotherapeutic when used alone.

(Antagonistic Effect CI >1.0, Additive Effect CI = 1.0, Synergistic Effect CI <1.0).

Immunoblot assessment of SG- and chemotherapy-mediated cell signaling *in vitro*

Cells (HCC1806, DMS 53, 5637, and RT4) were plated overnight in 6-well plates. The following day, SG, a chemotherapeutic (carboplatin or cisplatin), or the combination of SG and a chemotherapeutic were added to appropriate wells for 24 h. Concentrations for each agent is shown in the figure. Cells were harvested, lysed in Ripa buffer containing protease/phosphatase inhibitor cocktail (Cell Signaling Technology, Danvers, MA, USA) and protein concentrations were determined using BCA Protein Assay Kit (Thermo Fisher; Grand Island, NY, USA). A total of 20 mg protein was resolved in 4–12% Bis-Tris NuPAGE gels (Thermo Fisher Scientific; Cat. No. NP0322) and transferred to polyvinylidene difluoride (PVDF) membranes. Blots were blocked with 5% nonfat milk in 1x TBS-T for 1 h at room temperature. Membranes were probed overnight at 4°C with primary antibody, followed by 1 h incubation at room temperature with secondary antibody. Rabbit anti-human primary antibodies were purchased from Cell Signaling Technology and included anti-p21$^{waf1/cip1}$ (Cat. No. 2947s), anti-Cyclin D1 (Cat. No. 4967s), anti-Mcl-1 (Cat. No. D35A5), anti-survivin (Cat. No. 2803s) and anti-β-actin (Cat. No. 4967). Murine anti-human primary antibodies were purchased from Santa Cruz Biotechnology, Inc. (Dallas, TX, USA) and included anti-Bcl-2 (Cat. No. sc-7382) and anti-Bax (Cat. No. sc-20067). Horseradish peroxidase-conjugated secondary antibodies against rabbit (Cat. No. 7074s) and murine (Cat. No. 7076s) primary antibodies were purchased (Santa Cruz Biotechnology, Inc.). Thermo Fisher Scientific SuperSignal West Dura Extended Duration Substrate (Cat. No. F00041) was used for detection.

In vivo therapeutic studies

All animal studies were approved by Montclair State University Institutional Animal Care and Use Committee (Montclair, NJ). Mice set up with HCC1806 or 5637 tumor xenografts utilized 5-week old NCr female athymic nude (*nu*/*nu*) mice (Taconic Farms, Germantown, NY). Xenografts were established by harvesting cells from tissue culture and mixing 1:1 with matrigel, such that each mouse received a total of 1×10^7 cells s.c. in the right flank. For the DMS 53 xenografts, 5-week-old female C.B.-17 SCID mice (Taconic Farms) were utilized to establish tumors in their right flank. Mice were injected s.c. with 200 µL of DMS 53 tumor suspension (20% w/v) plus cells (5×10^6 cells per mouse) mixed 1:1 with matrigel. Tumor volume (TV) was determined by measurements in two dimensions using calipers, with volumes defined as: $L \times w^2/2$, where L is the longest dimension of the tumor and w the shortest. Mice were randomized into treatment groups and therapy begun when tumor volumes were approximately 0.3 cm^3. Treatment regimens, dosages, and number of animals in each experiment are described in the *Results* and in the Figure Legends. The lyophilized SG and control ADC (h679-CL2A-SN-38) were reconstituted and diluted as required in sterile saline. Likewise, all chemotherapeutics were diluted in sterile saline to achieve desired concentrations prior to administration to animals.

SUPPLEMENTARY REFERENCES

1. Reynolds CP, Maurer BJ. Evaluating response to antineoplastic drug combinations in tissue culture models. Methods Mol Med. 2005; 110:173–83. https://doi.org/10.1385/1-59259-869-2:173. [PubMed]

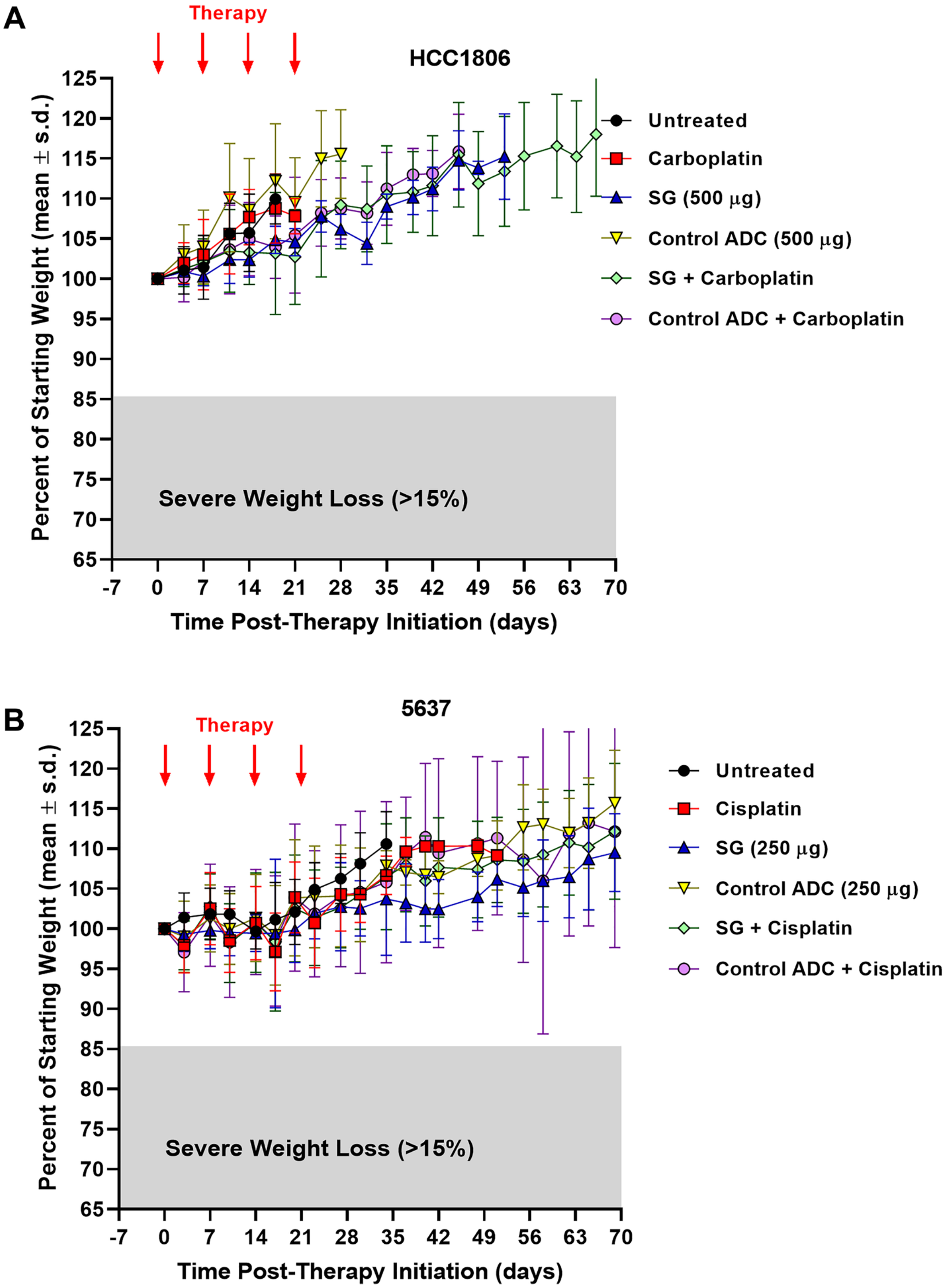

Supplementary Figure 1: Tolerability of SG combined with carboplatin or cisplatin in mice bearing human TNBC and UBC xenografts. Toxicity was assessed by changes in body weight in the animals treated with SG plus various combinations of (**A**) carboplatin in HCC1806 TNBC tumor-bearing mice, or (**B**) cisplatin in 5637 UBC tumor-bearing animals. Shaded area indicates weight loss greater than 15% (i.e., weight drop to 85% or lower of starting weight).

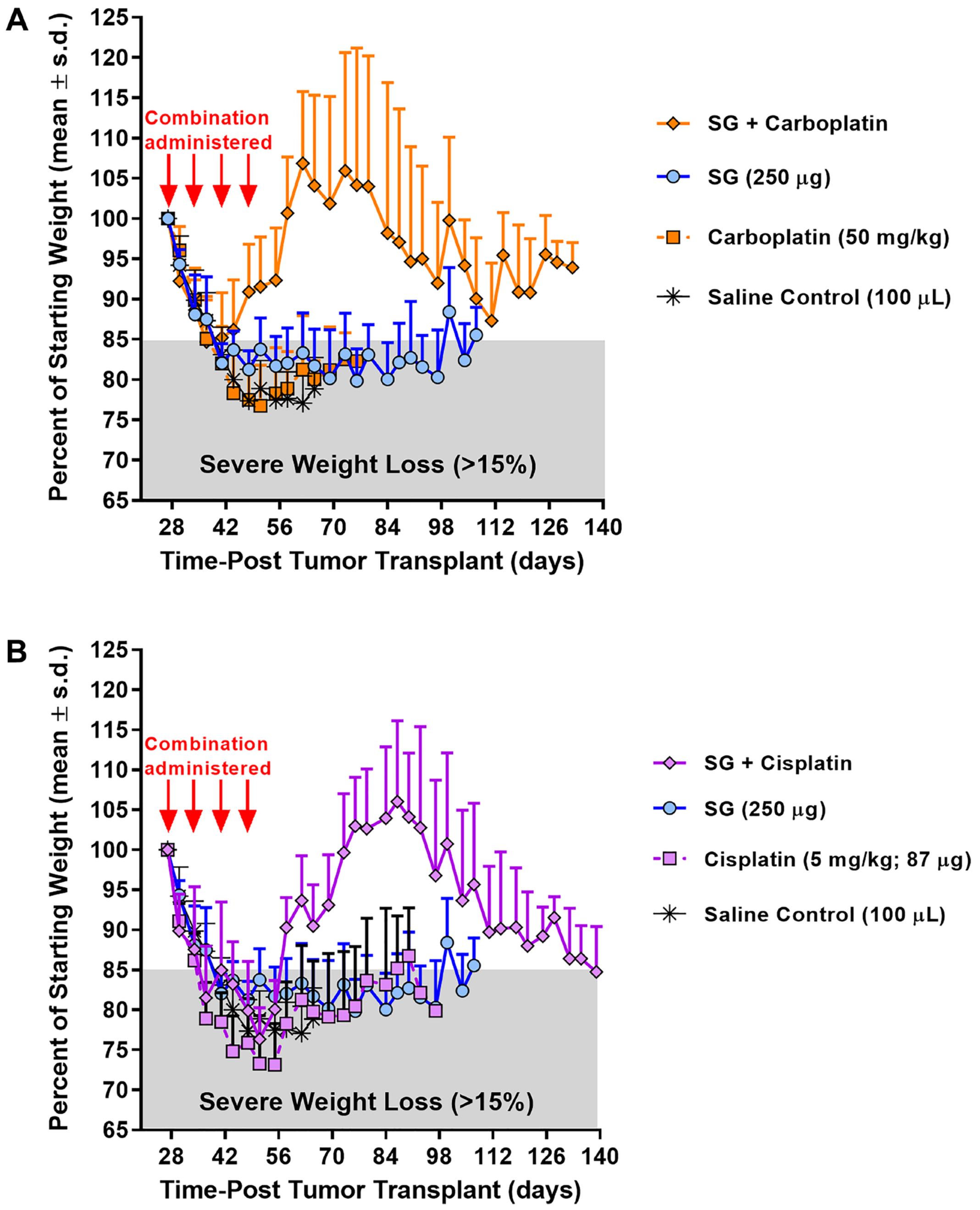

Supplementary Figure 2: Combination of SG plus carboplatin or cisplatin in mice bearing DMS 53 tumor-xenografts reverses cachexia. Animals were set up with DMS 53 tumor xenografts as described in Materials and Methods. Mice were treated with SG, carboplatin, cisplatin, or combinations at the indicated doses. Animals were weighed twice weekly and changes in whole body weights relative to the time the mice received their first treatment was calculated with 100% representing this starting weight. Since some mice were lost due to disease progression over time, the graphed data represents all surviving mice within a treatment group at the various time-points. (**A**) Tumor-bearing mice treated with the combination of SG plus carboplatin. (**B**) Mice treated with SG plus cisplatin. The red arrows in both graphs represented the days when the combination therapy was administered to the animals. As these data were from a single study, the SG monotherapy and saline control graphs are from the same mice in both (A) and (B). Separate graphs for carboplatin and cisplatin combinations with SG are presented for clarity.

Supplementary Table 1: Trop-2 surface expression levels in various solid tumor lines via FACS analysis*

Cell line	Tumor type	Surface Trop-2 Molecules per cell (Mean ± s.d.)
RT4	Urinary Bladder; Transitional Cell Papilloma	354,641 ± 36,904
5637	Urinary Bladder; Grade II Carcinoma	161,765 ± 6,014
T24	Urinary Bladder; Transitional Cell Carcinoma	78,206 ± 19,463
UM-UC-3	Urinary Bladder; Carcinoma	2,198 ± 921
DMS 53ǂ	Small Cell Lung Carcinoma	43,620 ± 4,557
Raji (negative control)	NHL	766 ± 35

*Three separate assays were performed on each cell line. ǂResults based on two separate assays. Abbreviation: s.d.: standard deviation.

Supplementary Table 2: Area under the curve comparisons between SG plus platinum-based chemotherapeutics versus controls in mice bearing various human tumor xenografts

Disease (cell line)	Combination	Control Treatment	Time of Comparison[a]	Tumor Volumes[b] (cm^3) (mean ± s.d.)	*P*-value[c]	*P*-value[d] (AUC)
TNBC (HCC1806)	SG (500 µg) *plus* Carboplatin vs.	SG (500 µg)	day 42	0.129 ± 0.034 vs. 0.765 ± 0.295	0.0005	0.0006
		Control ADC (500 µg)	day 18	0.157 ± 0.023 vs. 0.801 ± 0.253	0.0002	0.0003
		Carboplatin	day 7	0.245 ± 0.069 vs. 0.610 ± 0.287	0.0082	0.0062
		Control ADC *plus* Carboplatin	day 42	0.129 ± 0.034 vs. 0.881 ± 0.193	<0.0001	<0.0001
		Saline	day 11	0.229 ± 0.043 vs. 0.844 ± 0.322	0.0011	0.0001
TNBC (HCC1806)	SG (250 µg) *plus* Carboplatin vs.	SG (250 µg)	day 28	0.236 ± 0.126 vs. 0.738 ± 0.305	0.0039	0.0047
		Carboplatin	day 7	0.279 ± 0.046 vs. 0.610 ± 0.287	0.0134	0.0363
		Saline	day 11	0.269 ± 0.067 vs. 0.844 ± 0.322	0.0015	<0.0001
SCLC (DMS 53)	SG (250 µg) *plus* Carboplatin vs.	SG	day 46	0.282 ± 0.153 vs. 0.745 ± 0.162	<0.0001	0.0001
		Carboplatin	day 14	0.166 ± 0.019 vs. 0.602 ± 0.224	0.0004	0.0017
		Saline	day 14	0.166 ± 0.019 vs. 0.683 ± 0.185	<0.0001	0.0001
UBC (5637)	SG (250 µg) *plus* Cisplatin vs.	SG	day 76	0.239 ± 0.188 vs. 0.569 ± 0.482	0.0671	0.0362
		Control ADC	day 17	0.262 ± 0.141 vs. 0.645 ± 0.213	0.0002	0.0014
		Cisplatin	day 23	0.210 ± 0.094 vs. 0.812 ± 0.263	<0.0001	0.0030
		Control ADC *plus* Cisplatin	day 27	0.221 ± 0.072 vs. 0.542 ± 0.315	0.0081	0.0007
		Saline	day 17	0.262 ± 0.141 vs. 0.700 ± 0.162	<0.0001	<0.0001
SCLC (DMS 53)	SG (250 µg) *plus* Cisplatin vs.	SG	day 46	0.112 ± 0.026 vs. 0.745 ± 0.162	<0.0001	<0.0001
		Cisplatin	day 46	0.112 ± 0.026 vs. 0.758 ± 0.224	<0.0001	<0.0001
		Saline	day 14	0.156 ± 0.036 vs. 0.683 ± 0.185	<0.0001	<0.0001

[a]Time of comparison for AUC was from the day therapy was initiated up to the day the first animal in the control treatment group was lost due to disease progression as described in Materials and Methods. This is also the time when direct comparisons of final tumor volumes were made. [b]Mean tumor volumes for SG combination therapy group *versus* the various control groups on the last day mice were evaluable (i.e., the day post-therapy the first animal in that control group was lost). [c]Comparison of mean tumor volumes on the last day they were evaluable. [d]Overall tumor growth curve AUC comparison.
